# Severe Thrombocytopenia in Pregnancy Secondary to Influenza A

**DOI:** 10.7759/cureus.78449

**Published:** 2025-02-03

**Authors:** Donya T Sabet, Luke Prior, Lilantha Wedisinghe

**Affiliations:** 1 Department of Obstetrics and Gynaecology, Ipswich Hospital, Brisbane, AUS; 2 Faculty of Medicine, Griffith University, Gold Coast, AUS; 3 School of Medicine, Bond University, Gold Coast, AUS; 4 Department of Internal Medicine, Ipswich Hospital, Brisbane, AUS

**Keywords:** immune thrombocytopenia, influenza a infection, thrombocytopenia in pregnancy, thrombocytopenia management, viral induced thrombocytopenia

## Abstract

Thrombocytopenia is a common finding in pregnancy, with the majority due to gestational thrombocytopenia. Gestational thrombocytopenia is an innocuous condition that rarely requires treatment. Immune thrombocytopenia accounts for only a minority of thrombocytopenia in pregnancy and can have serious implications, especially in the peripartum period, including restrictions on birth plans and complications such as postpartum haemorrhage and neonatal thrombocytopenia. We present a case of possible immune thrombocytopenia secondary to influenza A infection. The case involves a 19-year-old, gravida-2, para-1, who developed severe thrombocytopenia shortly after influenza A, with a nadir platelet count of 31 × 10^9^/L. The platelet count returned to normal levels after six days of oral prednisolone 25 mg. This case highlights the importance of a thorough workup to identify more serious conditions causing thrombocytopenia.

## Introduction

Thrombocytopenia, defined as a circulating platelet count of less than 150 × 10^9^/L, is a common finding in pregnancy, with a prevalence of 7% to 12% in all pregnancies [1]. Thrombocytopenia may be associated with systemic disorders, and there is a wide range of aetiologies. During pregnancy, the majority of thrombocytopenia is due to gestational thrombocytopenia (GT) [1]. GT is often mild, rarely leads to complications, and often does not require any treatment. Due to the high percentage of thrombocytopenia in pregnancy being harmless, clinicians may be falsely reassured to revert to surveillance. However, clinicians must keep a high index of suspicion so as not to overlook other underlying pathology that have greater clinical implications.

We present a case of possible immune thrombocytopenia (ITP) secondary to a viral illness, specifically influenza. ITP accounts for only 1% to 4% of thrombocytopenia in pregnancy and may lead to postpartum haemorrhage and neonatal thrombocytopenia [1,2].

## Case presentation

A 19-year-old, gravida-2, para-1, presented to the Maternity Day Assessment Unit at 35+0 weeks gestation due to concerns about per-vaginal fluid loss. Three days prior to the presentation, she was diagnosed with influenza A in the community. Upon presentation, her respiratory symptoms were mild and recovering. Preterm, pre-labour rupture of membranes was excluded. A routine full blood count (FBC) was taken at the time, revealing a platelet of 80 × 10^9^/L. Her discharge plan included a follow-up visit to the Antenatal Clinic to monitor platelets in the subsequent week.

The patient presented two days later with contractions and was admitted for monitoring of threatened preterm labour. Clinical findings were reassuring, and there were no signs of impending labour. However, her FBC showed a significant drop in platelets to 37 × 10^9^/L, and therefore, she remained an inpatient for further workup, and Obstetric Medicine input was requested.

She had no known personal or family history of blood clotting disorders, and there were no clinical findings to indicate bleeding. The main differential diagnoses considered were ITP and GT. Initial investigations did not support pre-eclampsia (PET), haemolysis, elevated liver enzymes and low platelet (HELLP) syndrome, or disseminated intravascular coagulopathy (DIC).

Further investigations all returned unremarkable. These included coagulation studies, autoimmune screening, viral screening, von Willebrand screening, nutritional screening and soluble fms-like tyrosine kinase receptor-1 (sFLT-1)/placental growth factor (PLGF) (Table 1).

**Table 1:** Investigations for workup of thrombocytopenia Ab: antibody, ANCA: antineutrophil cytoplasmic antibodies, Anti-dsDNA Ab: double-stranded deoxyribonucleic acid antibody, CMV: cytomegalovirus, EBV: Epstein-Barr virus, eGFR: estimated glomerular filtration rate, ENA: extractable nuclear antigen, HIV: human immunodeficiency virus, IgG: immunoglobulin G, IgM: immunoglobulin M, VWF: von Willebrand factor

	Investigation	Value	Interpretation	Normal Range
Routine Haematology	Haemoglobin	121 g/L	Normal	98-142 g/L
	Platelet Count	31 × 10^9^/L	Low	150-430 × 10^9^/L
	Erythrocyte Sedimentation Rate	55 mm/hour	Normal	<72 mm/hour
Coagulation Profile	International Normalised Ratio	0.8	Low	0.9-1.2
	Prothrombin Time	9 seconds	Normal	9-13 seconds
	Partial Thromboplastin Time	29 seconds	Normal	25-38 seconds
	Fibrinogen	6.5 g/L	High. Note levels expected to rise in pregnancy	1.7-4.5 g/L
	Lupus Anticoagulant Screen	Negative	-	-
	Factor 8 Level	1.31 units/mL	Normal	0.50-1.50 units/mL
	VWF Antigen	4.12 units/mL	High. Note levels expected to rise in pregnancy	0.50-2.00 units/mL
	VWF GP1bR	3.92 units/mL	High. Note levels expected to rise in pregnancy	0.50-2.00 units/mL
	VWF Collagen Binding	>2.00 units/mL	High. Note levels expected to rise in pregnancy	0.50-1.50 units/mL
Nutrition Screen	Vitamin B12	480 pmol/L	Normal	133-680 pmol/L
	Folate Level	49.1 nmol/L	Normal	>9 nmol/L
Chemistry	eGFR	>90 mL/minute/1.73m^2^	Normal	>90 mL/minute/1.73m^2 ^
	Creatinine	47 μmol/L	Normal	45-90 μmol/L
	Alanine Transaminase	16 unit/L	Normal	<34 unit/L
	Aspartate Transaminase	24 unit/L	Normal	<31 unit/L
	C-reactive Protein	84 mg/L	High	<5 mg/L
Urine	Urine Protein/Creatinine Ratio	18 g/mol	Normal	<30 g/mol
Endocrine	SFLT-1/PLGF ratio	150	Normal	2-158
Viral Screen	CMV IgG	Non-reactive	-	-
	CMV IgM	Non-reactive	-	-
	EBV IgG	Reactive	-	-
	EBV IgM	Non-reactive	No evidence of acute infection	-
	Parvovirus B19 IgG	Non-reactive	-	-
	HIV Antigen/Antibody Screen	Non-reactive	-	-
	Hepatitis B Surface Antigen	Non-reactive	-	-
	Hepatitis B Surface Antibody	<10 IU/L	No immunity to hepatitis B	-
	Hepatitis B Core Antibody Total	Non-reactive	-	-
	Hepatitis C Antibody IgG	Non-reactive	-	-
HLA Tissue Typing	C-ANCA	Negative	-	-
	P-ANCA	Negative	-	-
Immunology	ENA Screen	Negative	-	-
	Anti-dsDNA Ab	1 IU/mL	Normal	<7 IU/mL
	Anti-cardiolipin Ab IgG	188 CU	Normal	<20 CU
	Complement Component 3	1.23 g/L	Normal	0.90-1.80 g/L
	Complement Component 4	0.40 g/L	Normal	0.10-0.40 g/L
	Anti-beta 2 Glycoprotein 1 Antibodies	0 G units	Normal	<20 G units
	Anti-nuclear Ab	Negative	-	-

Foetal well-being was monitored with cardiotocography and ultrasound.

A consultation was made with the haematologists at a tertiary hospital. ITP was deemed to be the main differential. Platelets dropped to a nadir of 31 × 10^9^/L, and therefore, a daily regimen of oral prednisolone 25 mg was commenced, along with a course of oseltamivir for her influenza. The following day, after a single dose of prednisolone 25 mg, her platelets increased to 52 × 10^9^/L. 

Potential implications of thrombocytopenia during pregnancy, labour and delivery were discussed with the patient. Recommendations include avoidance of neuraxial intervention in cases of platelet count less than 70 × 10^9^/L, avoidance of foetal scalp electrode and foetal blood sampling. Preference for forceps over vacuum should instrumental delivery be indicated. Given that her platelet count improved, she was discharged with a plan to monitor her platelet count weekly and continue oral prednisolone until delivery.

At 37+1 weeks gestation, after six doses of prednisolone, her platelet count increased to 277 × 10^9^/L. Induction of labour was planned at 38+0 weeks gestation to take advantage of normal platelet count and to avoid protracted steroid use. She, however, presented to a birth suite with spontaneous rupture of membranes at 37+3 weeks, and labour was augmented. Her platelet count was stable at 236 × 10^9^/L. She requested an epidural, which was facilitated as it was no longer contraindicated. Her platelets were monitored six hourly whilst in labour and remained stable. She was delivered by an emergency caesarean section due to obstructed labour at full dilatation, presenting in a deflexed occiput posterior position. The caesarean was uncomplicated, with an estimated blood loss of 400 mL.

Intravenous tranexamic acid 1g was administered at the time of caesarean section and was recommended to continue for up to two weeks postpartum. She was discharged to her GP for further monitoring. 

## Discussion

A retrospective multi-centre study investigated 118 pregnancies with ITP, reporting a median platelet count nadir of 66 × 10^9^/L, with only 22% with a nadir of less than 30 × 10^9^/L [3]. This case report highlights a severe case of thrombocytopenia with a nadir platelet of 31 × 10^9^/L.

Influenza can directly cause thrombocytopenia by infection of the platelets by the virus particles, which subsequently reduces the quantity of sialic acids on the cell surface by the action of viral neuraminidase enzymes. Desialylated platelets are flagged for clearance via hepatocellular and macrophage pathways [4]. Other general mechanisms of thrombocytopenia in viral infections include inflammation-induced platelet consumption, cytokine-mediated myelosuppression and platelet sequestration via hypersplenism and phagocytosis in severe infections [5].

ITP is well known to be triggered by viral infections such as hepatitis and varicella. However, influenza is a rare cause of ITP, as demonstrated by a single case study in the literature relating to ITP in pregnancy secondary to influenza [6]. Features of this case that favour a diagnosis of ITP over non-specific viral-induced thrombocytopenia include the low platelet nadir of 31 × 10^9^/L in the absence of critical illness, as well as the robust response to oral corticosteroids.

As opposed to GT, which does not require active treatment, 15 to 35% of ITP in pregnancy requires treatment prior to delivery [1,7]. First-line management includes corticosteroids and intravenous immunoglobulin [8-10]. Corticosteroids are generally safe in pregnancy; however, they have well-known complications, such as hyperglycaemia, when used in high doses or for prolonged periods [11]. There is no consensus for optimal second-line treatments; however, in severe cases, immunosuppressants and immunotherapies have been utilised with significant complications reported [11]. 

Another important consideration of ITP is its potential significant impact on a patient’s birth plan. A platelet count of >20 × 10^9^/L is recommended for vaginal deliveries and >50 × 10^9^/L for caesarean delivery, which may impact a patient’s original timing of birth [12]. Furthermore, neuraxial intervention is not recommended if platelets are below 70 × 10^9^/L, limiting a patient’s analgesic options [12]. Lastly, unlike GT, ITP can lead to neonatal thrombocytopenia in up to 10 to 15% of neonates, with a rare complication of neonatal intracranial haemorrhage [12].

## Conclusions

ITP may have serious implications, especially in the peripartum period. However, recognition and pre-emptive treatment are crucial to avoid complications and alteration of birth plans, such as decisions regarding delivery modality and restricting the use of neuraxial analgesia. Postpartum complications include maternal haemorrhage and neonatal thrombocytopenia.

The diagnosis of thrombocytopenia in pregnancy should be supported by a workup of aetiology, followed by a discussion with the patient regarding subsequent implications to their birth plan.
